# Mr. Smith's Case of Iuversion of the Uterus

**Published:** 1801-12

**Authors:** William Smith

**Affiliations:** Member of the Corporation of Surgeons of London. Bideford


### *To the Editors of the Medical and Phyſical Journal.*

GENTLEMEN,

THAT our Profeſſion has of late years been enriched with a variety of eſſential improvements both in Operative Surgery and Pharmacy, no one who is not wilfully blind or egregiouſly ignorant will venture to deny; and nothing can tend ſo much to promote the continuation of its progreſs towards perfection, as the liberal communications of medical men, through your's, as well as through every other channel of public notoriety; and if truth be made the ground work of theſe publications, an immenſe volume of Natural Pathology will gradually be unfolded, and each ſucceeding year increaſe its pages, and add to its ſtock of valuable materials. By conſulting this real Materia Medica, the ſtudent and young practitioner will derive their knowledge from its pureſt ſource, that is, from the treatment of analogous caſes by their cotemporaries in practice; and to accompliſh this moſt deſirable plan, be it remembered that truth only is wanting; and ſuch is its intrinſic excellence, that it is equally valuable when wrapt in the coarſe garb of provincial inelegance as when adorned with all the drapery of refined and claſſic language.

In a practice of ſeventeen years, both in Surgery and Pharmacy, it has been my cuſtom to take notes of all caſes whoſe idioſyncraſies, mode of treatment, or termination, afforded any reaſonable grounds to hope that a ſubſequent peruſal of them would improve either my own practice or that of others; and during the late war, the office I have held of Surgeon Agent for ſick and wounded Seamen and Priſoners of War both in this and the adjacent ſea-ports, has conſiderably increaſed my collection. Theſe materials, conſiſting of plain narratives of caſes, with occaſional remarks, I intended to arrange and publiſh as a volume of medical facts, but the want of leiſure combined perhaps with a natural indolence have for ſeveral years prevented my putting this intention into execution, and your Journal offering now ſo pleaſing and extenſive a channel of communication with the public, it will afford me pleaſure to become your occaſional correſpondent, in preference to the more laborious plan I originally intended to adopt.

The following caſe being, I believe, very unfrequent, and requiring the immediate application of a man's beſt judgment as well as manual aſſiſtance, to avert impending death, and in which not a moment ſhould be loſt, I have ſelected it as my firſt firſt; and ſhould you deem it worthy a place in your Journal, the publication of it will oblige, your's, &c.

*Bideford, October* 21, 1801. WILLIAM SMITH,

Member of the Corporation of Surgeons of London.

---

### Case I. An Inversion of the Uterus.

In the autumn of the year 1799, Mrs. ———, a young married woman about twenty-four years of age, tall, extenuated, and of a delicate conſtitution, finding herſelf pregnant of her firſt child, requeſted my attendance on her as her accoucheur, at her houſe, about two miles from this town; but having for ſome years declined this part of the profeſſion, particularly in the country, and my aſſiſtant being deemed a good midwife, I repeatedly declined her ſolicitations. Her eldeſt ſiſter, however, dying in child-bed ſoon after, the violence of her apprehenſions increaſed to ſuch a degree, that I was induced, though reluctantly, to wave my objection, and promiſed to attend her.

Her labor commenced about four o'clock in the afternoon of the 9th of February, 1800; its progreſs was gradual, and in no reſpect differed from uſual natural labors. Her mind only underwent the moſt frightful apprehenſions; the recent death of her ſiſter agitated her beyond meaſure, and every approaching pain ſhe conſidered as the harbinger of her own diſſolution. In vain I tried to ſoothe her fears by aſſurances that ſhe was in the beſt poſſible way, and at no very diſtant time would be happily delivered; the recurrence of every throe renewed her alarm; and in this ſtate of agitation of mind, (at times almoſt amounting to agony) ſhe continued until three o'clock in the morning of the 10th, when ſhe was delivered of a fine boy.

When the labor drew near to a concluſion, and the head being elongated protruded in perinæo, I obſerved that it was retracted between each pain with unuſual violence, occaſioned, as I afterwards found, by the placenta being attached to the fundus uteri, and the extreme ſhortneſs of the funis, which after the excluſion of the child, created conſiderable embarraſſment in making the neceſſary ligatures.

Theſe circumſtances neceſſarily took up ſome time, during which ſhe had fainted; having delivered the child to the nurſe, I applied volatiles to the noſtrils, gave her ſome wine and water, and waited ſeveral minutes without making any effort to ſolicit the ſeparation of the placenta. Shortly after a violent pain called me to the bed-ſide; and upon touching the funis, (which had been conſiderably protruded ſince the ſeparation of the child) I found none of the uſual reſiſtance; and another pain succeeding, she exclaimed that her bowels were coming from her, and instantly an immense tumour was projected with considerable violence, ex vagina, and extended almost to her knees. A dreadful syncope immediately ensued, with subsultus tendinum, and a pulse scarcely perceptible.

Alarmed exceedingly, I began an examination of this singular phenomenon, which was of an oval form, and firm and fleshy in substance; when tracing the funis to the placenta, I found it attached to the apex of the tumor, which left me no room to doubt but that the whole uterus in a collapsed state (the usual contraction not having taken place after birth) was inverted and excluded from the body, and that the extreme point of the tumor, with the cake affixed thereto, was no other than the fundus uteri completely turned inside out.

Thus circumstanced, the remembrance of this infrequent and generally fatal case so impressively described by my worthy friend and teacher Dr. Lowder, in his admirable lectures, flashed upon my mind; and in conformity with his advice, without loss of time, I peeled of the placenta, and applying the back part of my hand with my fingers clenched to the apex or inverted fundus, I carried up my arm encircled in the double foldings of the uterus, per vaginam, until I had restored the uterus to its natural position; nor did I withdraw my arm immediately, which operating as an extraneous body, soon excited the uterus to action and produced a consequent reduction of its diameter.

By these means she was roused from her syncope; cloths wetted with cold vinegar were applied to the abdomen and pudenda, and she took tinct. opii gt. 40, in a glass of wine.

The hæmorrhage was moderate, but the subsultus and excessive quickness of the pulse continued for several days, and severe symptomatic fever came on, with a total inability of motion, the slightest elevation of the body producing faintness and an aggravation of all her symptoms.

On the 11th, she had urined freely; some perspiration was obtained by the aqua ammonia acetata combined with tinct. opii, and at night she slumbered a little.

On the 12th, a stool was procured by an enema, and a slight procidentia uteri, in its contracted state, took place; but the supine posture, and slight pressure by the nurse, soon reduced it.

From the 13th, the fever gradually abated, the milk was secreted, and she suckled her child six months, but her health has been very delicate ever since, occasioned by severe pains of the back and loins, and great weakness.

There appears to me to be an important distinction between this case, and that so well described by Mr. Welsh in your 27th Number.

Number. In Mr. Welſh's caſe, the uterus deſcended with, or immediately after the child, without pain or any ſeverity of ſymptoms; but in this caſe an interval of at leaſt ten minutes had elapſed after the child was born, and the inverſion was attended with a freſh expulſive excitement, and pain equally as powerful as if a ſecond child was to have been born.

*Quere.*—Did this extraordinary caſe ariſe merely from the poſition of the placenta and ſhort funis, neceſſarily dragging down the fundus uteri, when the child was born? Or, was not her whole frame prediſpoſed to a morbid atony, thereby occaſioning the want of the uſual contractile action of the uterus poſt partum from the operation of the mind?

